# Emotion and aging: evidence from brain and behavior

**DOI:** 10.3389/fpsyg.2014.00996

**Published:** 2014-09-09

**Authors:** Natalie C. Ebner, Håkan Fischer

**Affiliations:** ^1^Psychology, Social-Cognitive and Affective Development Lab, Department of Psychology, University of FloridaGainesville, FL, USA; ^2^Department of Psychology, Stockholm UniversityStockholm, Sweden

**Keywords:** emotional aging, brain-behavior links, cognition–emotion interactions, age-of-face effects, emotion regulation

## Abstract

Emotions play a central role in every human life from the moment we are born until we die. They prepare the body for action, highlight what should be noticed and remembered, and guide decisions and actions. As emotions are central to daily functioning, it is important to understand how aging affects perception, memory, experience, as well as regulation of emotions. The Frontiers research topic *Emotion and Aging: Evidence from Brain and Behavior* takes a step into uncovering emotional aging considering both brain and behavioral processes. The contributions featured in this issue adopt innovative theoretical perspectives and use novel methodological approaches to target a variety of topics that can be categorized into three overarching questions: *How do cognition and emotion interact in aging in brain and behavior? What are behavioral and brain-related moderators of emotional aging? Does emotion-regulatory success as reflected in brain and behavior change with age?* In this perspective paper we discuss theoretical innovation, methodological approach, and scientific advancement of the 13 papers in the context of the broader literature on emotional aging. We conclude by reflecting on topics untouched and future directions to take.

Emotions play a central role throughout human life. They highlight what is important and guide actions. As emotions are central to daily functioning, it is important to understand how aging affects them. An early scientific view argued that aging led to a deterioration of emotional function. This theory, represented by Carl Jung, claimed that old age is a period in life when people feel emotional sameness and the aging emotional landscape was described as bleached and barren. However, current psychological research shows that emotion is relatively unaffected by aging or even improves with age, in contrast to most cognitive functions (Scheibe and Carstensen, 2010). For example, aging is associated with improved emotional problem solving (Blanchard-Fields, 2007) and increased frequency of positive feelings (Carstensen et al., 2011). However, other emotional capacities, such as the ability to recognize other’s emotions, decline with age (Ruffman et al., 2008). Such variations across emotional functional domains, and our limited understanding of mediating and moderating factors, call for more research. Given various representational levels of the phenomenon, an approach that combines brain and behavior in uncovering emotional aging seems promising.

The current Frontiers research topic takes a step into this direction. It comprises novel theoretical and methodological approaches and presents exciting new findings. The contributions evolve around three broader questions that have only recently received attention in the literature on emotional aging and answers to which will inform affective science research: (1) *How do cognition and emotion interact in aging in brain and behavior?* (2) *What are behavioral and brain-related moderators of emotional aging?* (3) *Does emotion-regulatory success as reflected in brain and behavior change with age?* Within those overarching questions, different emotion-related phenomena (emotion perception, emotion experience, attention and memory related to emotion, emotion regulation) will be addressed.

In the following, we will summarize the contributions. We will illustrate how they increase scientific understanding of emotional aging. In particular, we will emphasize how adoption of diverse theoretical and methodological approaches allow for a multi-faceted study of emotional aging and we will lay out how adopting an aging brain-behavior prespective increases understanding of cognition–emotion interactions, moderators of emotion processing, and emotion-regulatory success. We will conclude by raising topics not yet covered for exploration in future research.

## NEW THEORETICAL AND METHODOLOGICAL APPROACHES IN THE STUDY ON EMOTION AND AGING

The research topic presents novel theoretical perspectives on emotional aging. Two of these are featured in perspective papers as summarized next. Other approaches are embedded in the context of the empirical paper contributions and integrated in the discussion below.

### A DISCRETE EMOTIONS APPROACH THAT CONSIDERS MULTI-DIRECTIONAL AGE DIFFERENCES IN SPECIFIC EMOTIONS OFFERS NEW DIRECTIONS OF RESEARCH

Complementing lifespan theories of developmental regulation that define negative affect and positive affect broadly (e.g., Baltes and Baltes, 1990), Kunzmann et al. (2014) propose a discrete emotions perspective on emotional aging. This approach links emotions to goals, emphasizing person–context interactions in their impact on adaptiveness of specific emotions (Haase et al., 2012). The approach is illustrated by delineating differential adult lifespan trajectories in the experience of anger and sadness. It sheds light on the mechanisms related to cognitive and physiological resources that underlie adaptive consequences of anger reactions in young adulthood and sadness reactions in old age and may prove particularly useful in understanding multi-directionality of affective responses across the adult lifespan. Extending this theoretical perspective to other emotions and across the entire lifespan will guide future research. English and Carstensen (2014) and Svärd et al. (2014) in this issue adopted a discrete emotions perspective. In contrast, Völkle et al. (2014) promote the usefulness of a multi-dimensional emotions approach, proposing that more than just one emotion is represented in a face. Other contributions differentiate broadly between positive and negative emotions (Pehlivanoglu et al., 2014; Petrican et al., 2014; Truong and Yang, 2014) and/or highlight the impact of the emotion dimension of arousal (Dolcos et al., 2014; English and Carstensen, 2014; Svärd et al., 2014; Truong and Yang, 2014).

### AGE OF THE FACE AFFECTS INTERPRETATION OF FACIAL EXPRESSIONS ACROSS THE ADULT LIFESPAN

The ability to read facial emotions in others declines with age (Ruffman et al., 2008). Fölster et al. (2014) propose that beyond effects of the age of the observer, effects of the age of the face, in interaction with the emotion expressed in the face, need to be considered in research on facial emotion perception. In particular, group differences in expressive style, higher familiarity with faces of in-group members (Elfenbein and Ambady, 2002) and increased motivation toward in-group faces (Thibault et al., 2006) may contribute to age-congruency effects. Fölster et al. (2014) importantly conclude that such effects are crucial in the context of face memory (Rhodes and Anastasi, 2012) but may play less of a role in facial emotion perception. The proposed perspective will facilitate future examination of how age stereotypes influence face recognition bias and how age differences in the frequency of experiencing certain emotions may affect change in facial features. Use of longitudinal approaches and ecologically valid stimuli, such as implemented in some contributions in this issue (Petrican et al., 2014; Riediger et al., 2014), appear particularly promising.

This issue is characterized by a wide selection of methodological approaches, reflecting the complexity of the emotional aging phenomenon. Employed approaches are experience sampling (English and Carstensen, 2014), subjective evaluations (Petrican et al., 2014; Riediger et al., 2014; Svärd et al., 2014; Völkle et al., 2014), cognitive-behavioral measures (Pehlivanoglu et al., 2014; Svärd et al., 2014; Truong and Yang, 2014), eye tracking (Pehlivanoglu et al., 2014), functional neuroimaging (Allard and Kensinger, 2014; Cassidy et al., 2014; Dolcos et al., 2014; Opitz et al., 2014), and electrophysiology (Opitz et al., 2014). Some of the contributions apply multiple methods to the same sample (Opitz et al., 2014; Pehlivanoglu et al., 2014), enabling integration of research findings. However, this research topic, as is characteristic of the current research field, also faces methodological heterogeneity between studies. While this allows for a multi-faceted reflection on emotional aging, a direct comparison across studies is difficult. Innovatively, several contributions leverage new statistical advancements in multi-level modeling to decompose intra-individual from inter-individual variability (English and Carstensen, 2014; Opitz et al., 2014; Petrican et al., 2014).

## COGNITION–EMOTION INTERACTIONS IN AGING FROM A BRAIN-BEHAVIOR PERSPECTIVE

A growing number of studies are targeting cognition–emotion interactions. The majority of these studies examine behavioral age-related change (Isaacowitz and Riediger, 2011). Still little is known about the cognition–emotion interplay from an aging brain perspective (Fischer et al., 2010; Samanez-Larkin and Carstensen, 2011). Various contributions in this issue have addressed this research gap. As summarized next, Völkle et al. (2014) demonstrate a mood-emotion perception link across the adult lifespan. Svärd et al. (2014) show direct effects of emotion evaluations on emotion-related cognition. Cassidy et al. (2014), Pehlivanoglu et al. (2014), and Truong and Yang (2014) clarify age differences in working memory/source memory for information with emotional content.

### MOOD INFLUENCES YOUNG AND OLDER ADULTS’ EMOTION PERCEPTION AND EMOTION PERCEPTION IN TURN AFFECTS MOOD

In Völkle et al. (2014) young, middle-aged, and older adults indicated their current mood before providing facial ratings along prototypical emotional expressions, using a multi-dimensional approach to emotions. Crossed-random effects analyses supported a mood-congruency effect: after controlling for accurate recognition of the primary facial expression, better mood increased the likelihood of perceiving additional facial happiness, while it reduced the likelihood of perceiving additional negative facial expressions. A reversed pattern held for negative mood. These effects were primarily shown by older adults. By assessing naturally occurring fluctuations in mood this study addresses cognition–emotion interactions in aging in a more ecologically valid way than experimental mood manipulation studies typically conducted in this domain.

### YOUNG AND OLDER ADULTS DIFFER IN SUBJECTIVE RATINGS OF EMOTIONAL FACES, WITH EFFECTS ON ATTENTION AND MEMORY FOR FACES

Svärd et al. (2014) adopted a three-dimensional approach to emotions by considering young and older adults’ facial ratings of valence (pleasant/unpleasant), arousal (active/passive), and potency (weak/strong; Keil and Freund, 2009). They observed an age-related flattening of subjective impressions of facial emotions. Regression analyses confirmed a direct link between subjective ratings and task performance in that higher potency (but not arousal and valence) ratings of angry faces predicted better attention and memory for faces. This work contributes to the sparse knowledge on the interplay between subjective emotion ratings on emotion-related cognition in aging.

### EMOTIONAL INFORMATION BOTH FACILITATES AND DISRUPTS WORKING MEMORY IN AGING

Emotional content of information should affect working memory pronouncedly in older adults, given increased emotion orientation (Carstensen, 2006) and preserved emotion processing (Ebner et al., 2012) with age. Using a working memory paradigm for target information in the presence of distraction, Truong and Yang (2014) systematically varied valence and arousal of word stimuli. For both age groups emotional targets facilitated working memory, while emotional distracters disrupted performance. Emotional disruptive effects were limited to negative words and occurred only in older adults. By identifying situations in which older adults’ preserved emotional processing as a helpful “friend” versus a hindering “foe” for cognition, this work adds to the growing literature on the emotion-cognitive control interplay in aging (Dolcos et al., 2011). It supports recent frameworks on competitive advantage of emotional information in aging (Carstensen, 2006) and the role of goal relevance of emotion within specific task contexts (Pessoa, 2008).

### AGING IS ASSOCIATED WITH A DEFICIT IN UNBINDING IRRELEVANT EMOTIONAL INFORMATION FROM MEMORY

Pehlivanoglu et al. (2014) confirmed an age-related hyper-binding hypothesis according to which older compared to young adults show increased binding of task-irrelevant information (Campbell et al., 2010). This age-related deficit in unbinding task-irrelevant facial emotion information held beyond age-related differences in perception, attention, or short-term memory. Innovatively, the study employed pupil dilation and showed greater cognitive resource recruitment during attentional processing (Goldinger and Papesh, 2012) in older than young adults. Addition of neuroimaging data on the brain locus of the observed effects will further unwind the link between emotion and working memory in aging.

### AGE DIFFERENCES IN ENCODING OF SOURCE INFORMATION ARE AMELIORATED FOR SOCIOEMOTIONAL INFORMATION

In Cassidy et al. (2014) young and older adults encoded statements that varied in perceived truth value, as a type of socioemotional information. In line with work suggesting that socioemotional information reduces age-related source memory deficits (Cassidy and Gutchess, 2012), there was an age-related increase in encoding-related ventral relative to dorsal mPFC recruitment in older compared to young participants. This work importantly contributes to age-differential mPFC function in emotion-related source memory and suggests an increased focus on processing of emotionally relevant information, as opposed to knowledge acquistion, in aging.

## MODERATORS OF EMOTIONAL AGING FROM A BRAIN-BEHAVIOR PERSPECTIVE

Mechanisms underlying age deficits in the ability to read emotions in others are not well understood yet. The literature discusses age-related change in visual processing, brain structure and function, hormones, and neurotransmitters as possible explanations (Ruffman et al., 2008; Ebner et al., 2013). Recently, moderating factors such as arousal, emotion expressed, and face-age have received attention (Fölster et al., 2014). Accordingly, papers in this issue have taken up investigation of such moderating factors. As laid out next, Riediger et al. (2014) show moderator roles of age-of-face and genuineness of emotion in emotion perception. Studying proficiency in emotion perception as predictor of well-being, Petrican et al. (2014) provide evidence for a moderating function of neural degenerative disease. English and Carstensen (2014) demonstrate how specific emotions, variations in arousal, and variations in time of day moderate everyday life emotion experience.

In response to critique that the majority of studies on emotion perception use photographs of prototypic facial expressions (Isaacowitz and Stanley, 2011) and examine emotion in the artificial lab setting, several contributions in this issue increased the ecological validity of their stimuli by using dynamic emotion expressions (Riediger et al., 2014) and whole-body postures (Petrican et al., 2014), and by assessing emotion in everyday life (English and Carstensen, 2014) and with relevance in clinical-dyadic context (Petrican et al., 2014).

### YOUNG AND OLDER ADULTS DIFFER IN THEIR ABILITY TO IDENTIFY EMOTIONAL EXPERIENCES ACCOMPANYING SMILES, WITH VARIATIONS BY GENUINENESS OF SMILES AND AGE OF THE SMILING PERSON

People show similar facial expressions in disparate situations. Riediger et al. (2014) developed an extensive set of dynamic video episodes of positive-affective, negative-affective, and affectively neutral smiles of young and older adults. Contrasting previous work (Murphy et al., 2010), young participants outperformed older participants at identification of emotional experiences accompanying smiles. This improved performance in young relative to older adults was attenuated for older faces. Older adults were less likely than young adults to attribute positive emotions to smiles, and more likely to indicate a smile as posed. However, young adults more frequently attributed positive emotions to smiles in older than young faces. Use of dynamic, content-valid smile expressions provide a promising venue for studying age differences in emotion recognition and consideration of age-of-face moderation further informs the picture.

### PARTNER’S PROFICIENCY IN IDENTIFYING POSITIVE VERSUS NEGATIVE EMOTIONS IN OTHERS DIFFERENTIALLY PREDICTS WELL-BEING IN PARKINSON’S DISEASE (PD) PATIENTS VERSUS NEUROLOGICALLY INTACT AGE-MATCHED CONTROLS

Parkinson’s disease is associated with reduced emotional expressivity (Simons et al., 2004). In a clinical-dyadic context, Petrican et al. (2014) used a pointlight walker paradigm that depicted emotions in whole-body postures. Spouses of PD patients were better in identifying positive emotions but worse in identifying negative emotions. Also, relative to controls, they underestimated the intensity in negative emotions, possibly as a compensatory mechanism. Greater expertise in identifying positive emotions was linked to greater spousal well-being in healthy elderly couples; for PD patients and their spouses greater proficiency in reading negative emotions predicted greater spousal well-being. This study is one of the few to shed light on the link between emotion recognition proficiency and close partner well-being in an intimate partnership (Gable et al., 2012), suggesting a moderating role of neurological status. The dynamic whole-body emotional stimuli were more naturalistic than the static facial emotional stimuli typically employed in this research field. Further scientific advancement will come from future implementation of longitudinal dyadic studies to assess emotion expressive habits of both spouses, their emotion expertise, and well-being over time.

### AGE DIFFERENCES IN EVERYDAY LIFE EMOTIONAL EXPERIENCE VARY ACROSS EMOTIONS AND TIME OF DAY

English and Carstensen (2014) used experience sampling to assess emotional experience twice a day across 10 days in community-dwelling young and older adults. Both age groups felt less positive and more negative in the evenings than in the mornings. Older compared to young adults reported relatively more positive emotions at both times of day. They also reported greater experience of positive emotions with the exception of the two high-arousal emotions excitement and pride. In contrast, age-related reductions in negative experience were observed only for reports of low-arousal negative emotions. For some emotions (relaxed) age differences were stronger in the mornings, whereas for other emotions (enthusiastic) age differences were more pronounced in the evenings. These findings underscore the importance of examining emotional aging in everyday life, thereby carefully considering the moderating roles of emotions sampled and assessment time.

## EMOTION-REGULATORY SUCCESS IN AGING FROM A BRAIN-BEHAVIOR PERSPECTIVE

Emotion regulation refers to promotion of helpful emotions while managing harmful emotions (Gross, 2013). Appropriately regulating one’s feelings in favor of successful social interactions may become particularly relevant in old age, given increased dependency and social loss. There is behavioral evidence that older compared to young adults show improved emotion-regulatory capacity (Urry and Gross, 2010). Despite normative declines in various functional domains, improved emotion-regulatory capacities may contribute to high levels of life satisfaction in aging [English and Carstensen (2014) for qualification of these findings]. In contrast, neuroimaging evidence suggests that brain regions characterized by age-related decline in volumetric gray matter (Raz et al., 2004) are relevant for successful emotion regulation (Buhle et al., 2013). As summarized next, age-related change in emotion-regulatory success in brain and behavior were examined across three studies. Allard and Kensinger (2014) demonstrate age differences in efficient use of cognitive reappraisal. Dolcos et al. (2014) show emotion-regulatory benefits of spontaneous recruitment in emotion control regions in aging. Opitz et al. (2014) describe variations in emotion-regulatory success as a function of fluctuating resources across adulthood.

### OLDER COMPARED TO YOUNG ADULTS USE EMOTION-REGULATORY STRATEGIES LESS EFFICIENTLY

Allard and Kensinger (2014) engaged young and older adults in emotion-regulatory strategies in response to negative film clips. When comparing regulation (selective attention, cognitive reappraisal) to passive viewing, young adults showed greater regulation-related activity in lateral and medial PFC while older adults showed greater dorsolateral PFC activity. Activity in dorsolateral PFC was increased for reappraisal compared to selective attention in older but not young adults, possibly reflecting a compensation for less efficient cognitive control processing in aging. Consistent with this interpretation, the timing of reappraisal-related activity in ventrolateral PFC was delayed for older adults. This research constitutes a first step toward identification of emotion-regulatory strategies that are effective in aging. The authors emphasize adoption of trial-by-trial approaches to determine success for particular emotion-regulatory attempts as fruitful for future brain-behavior investigations.

### OLDER COMPARED TO YOUNG ADULTS SPONTANEOUS ENGAGEMENT OF EMOTION CONTROL BRAIN REGIONS RESULTS IN EMOTION-REGULATORY BENEFITS

Low-arousing negative stimuli engage controlled processes (Kensinger and Corkin, 2004), while high-arousing information captures attention automatically (Dolan, 2002), a process preserved in aging (Mather and Knight, 2006). In Dolcos et al. (2014) young and older participants viewed emotional pictures, that varied in arousal, and rated them for emotional content. Variations in amygdala and ventromedial PFC activity suggested that older adults engaged more automatic processes when evaluating high-arousing negative information, and more controlled processes in response to low-arousing negative information. Linking brain and behavior, spontaneous engagement of emotion control regions reduced subjective experience of low-arousing negative information in older adults, supporting the idea of chronic activation of emotion regulation in aging and delineating neural correlates underlying enhanced emotional well-being in aging.

### FLUID COGNITIVE ABILITY INCREASES EMOTION-REGULATORY SUCCESS IN YOUNG AND OLDER ADULTS

Successful cognitive reappraisal recruits brain areas involved in working memory (McRae et al., 2010) and is most effective when initiated early in the emotion-generative cycle (Sheppes and Meiran, 2007). Consequently, age-associated decline in fluid cognitive abilities should negatively impact cognitive reappraisal success. Opitz et al. (2014) showed that both young and older participants reinterpreted the meaning of sad pictures (versus passive viewing). Emotional responding was measured using a multiple-channel approach that integrated self-reported emotional intensity, expressive behavior, and autonomic physiology. Multi-level modeling showed that fluid (but not crystallized) cognitive abilities predicted emotion-regulatory success, independent of age. The research importantly supports the role of fluctuating resources across adulthood on emotion-regulatory success on brain-behavior levels.

## OPEN QUESTIONS AND FUTURE DIRECTIONS

The work discussed in this perspective paper provides novel insights into the study of emotional aging, providing responses to pressing research questions. However, various topics remain untouched, offering opportunities for exciting future research moving forward in this domain of inquiry. All articles in this issue adopted a cross-sectional approach. Longitudinal, lifespan research will allow examination of gradual quantitative and qualitative emotional change over the life-cycle, allowing to draw a comprehensive picture of emotional development. With one exception (Petrican et al., 2014), all papers used community-dwelling older research participants, prescreened to be free of serious affective or cognitive impairments. A promising future avenue is emotional aging research in clinical contexts such as in dementia, apathy, or social anxiety, pathologies with high relevance in aging (Goodkind et al., 2010). Expanding current research to more diverse samples coupled with continuous use of advanced methodology will move forward this emerging field. A thorough investigation of consequences of age-related emotional change on health and quality of social interactions is currently missing. Petrican et al. (2014) started exploring this territory and demonstrated an association between emotion recognition proficiency and well-being in elderly couples. Relatedly, a stronger research focus toward improvement of emotional aging is warranted such as through administration of medicinal products (Ebner et al., 2013; Campbell et al., 2014) or training of volitional brain activation associated with emotion-regulatory success (Caria et al., 2010). Several of the papers reflect a desirable development toward integration of positive and negative stimulus material (English and Carstensen, 2014; Riediger et al., 2014; Svärd et al., 2014; Völkle et al., 2014). Also, crucial to further advancement of the multi-faceted phenomenon of emotional aging will be an integration of brain-behavior links, thereby considering hormonal, genetic (Ebner et al., 2013) and contextual, motivational change (Carstensen, 2006). We look forward to integrative research advancements in this exciting domain.

## Conflict of Interest Statement

The authors declare that the research was conducted in the absence of any commercial or financial relationships that could be construed as a potential conflict of interest.
